# Metabolic Syndrome, Cardiovascular Risk, and Health-Related Quality of Life Among Police Officers in Madeira: A Cross-Sectional Occupational Health Study

**DOI:** 10.3390/healthcare14060751

**Published:** 2026-03-17

**Authors:** Jerónimo Pina, Vanessa Santos, Luís Miguel Massuça

**Affiliations:** 1 Polícia de Segurança Pública, 1199-010 Lisbon, Portugal; jeronimopina@gmail.com; 2 CIPER, Faculty of Human Kinetics, University of Lisbon, 1495-751 Cruz Quebrada, Portugal; vanessa.santos@ipiaget.pt; 3 Insight: Piaget Research Center for Ecological Human Development, Instituto Piaget, 2805-059 Almada, Portugal; 4 ICPOL—Police Research Center, Higher Institute of Police Sciences and Internal Security, 1300-352 Lisbon, Portugal; 5 CIFI2D, Faculty of Sport, University of Porto, 4200-450 Porto, Portugal; 6 CIDEFES, Faculty of Physical Education and Sport, Lusófona University, 1749-024 Lisbon, Portugal

**Keywords:** cardiovascular risk, Framingham risk score, law enforcement, metabolic syndrome, occupational health, police officers

## Abstract

**Highlights:**

**What are the main findings?**

- Metabolic syndrome (MetS) prevalence among police officers (POs) was 28.4%.
- Male POs showed significantly higher Framingham Cardiovascular Risk scores than female POs.
- Approximately 20% of POs were classified as high cardiovascular risk.
- No significant sex differences in MetS prevalence were observed.
- Male POs performing indoor duties presented higher cardiovascular risk scores.

**What are the implications of the main findings?**

- POs represent a priority occupational group for systematic cardiometabolic screening due to their increased cardiovascular vulnerability.
- Workplace health promotion strategies, including smoking cessation and structured physical activity programs, are particularly warranted for male POs in indoor roles.

**Abstract:**

**Background/Objectives**: Police work has been associated with increased cardiometabolic risk due to occupational stress, shift work, and lifestyle factors. This study aimed to determine the prevalence of MetS and 10-year cardiovascular risk, and to analyse differences by sex and occupational function among police officers (POs) in Madeira. **Methods**: A cross-sectional study was conducted among 109 POs from the Autonomous Region of Madeira. MetS was defined according to the National Cholesterol Education Program Adult Treatment Panel III (NCEP ATP III) criteria, and 10-year cardiovascular risk was estimated using the Framingham Risk Score. Health-related quality of life (HRQoL) was evaluated using the SF-36 questionnaire (SF-36v2). Comparisons were performed by sex and professional role (indoor *versus* outdoor). **Results**: (i) The prevalence of MetS was 28.4%; (ii) Male POs had significantly higher Framingham Risk Scores than female POs, although no sex differences in MetS prevalence were observed; (iii) Approximately 20% of POs were classified as high cardiovascular risk; and (iv) Among male POs, those performing indoor duties showed higher cardiovascular risk scores. **Conclusions**: POs in Madeira present a considerable burden of cardiometabolic risk factors. These findings highlight the need for targeted occupational health strategies and regular cardiovascular screening within police organisations.

## 1. Introduction

Cardiovascular diseases (CVDs) remain the leading cause of morbidity and mortality worldwide, accounting for an estimated 17.9 million deaths each year [1]. In Portugal, CVD are responsible for approximately one-third of all deaths, representing a significant burden on the healthcare system and the working population [2]. This highlights the importance of identifying, monitoring, and addressing the major modifiable risk factors that contribute to their development. Among these, physical inactivity, hypertension, overweight, smoking, diabetes mellitus, and hypercholesterolemia stand out as key contributors to cardiovascular risk. Many of these factors are also directly associated with the diagnosis of Metabolic Syndrome (MetS) and with the estimation of cardiovascular risk using tools such as the Framingham Risk Score, both of which are widely used in clinical and epidemiological research [3].

MetS represents a cluster of interrelated metabolic abnormalities, including abdominal obesity, insulin resistance, hypertension, dyslipidaemia, and elevated fasting glucose, that synergistically increase the risk of type 2 diabetes mellitus and CVD [3,4]. The presence of three or more of these components defines the condition and serves as an early warning of future cardiometabolic disease [3,4]. Beyond its clinical significance, MetS is also a public health concern, given its association with sedentary lifestyles, poor diet, and occupational stress, all factors that have become increasingly prevalent in modern societies [5]. Complementary to this, the Framingham Risk Score, derived from the historic Framingham Heart Study, provides an integrative approach to quantifying the 10-year probability of developing a cardiovascular event [6]. Based on variables such as age, sex, blood pressure, lipid profile, diabetes, and smoking status, the model remains one of the most validated tools for cardiovascular risk prediction in population studies [6,7].

Police work, while essential to public safety, is recognised as one of the most demanding and stressful professions, combining physical danger, irregular schedules, and chronic psychosocial stress [8,9]. The unique occupational environment of law enforcement exposes police officers (POs) to multiple risk factors that may compromise their long-term health [9,10]. Extended working hours, shift rotations, sleep deprivation, and exposure to potentially traumatic events contribute to autonomic dysregulation, hormonal imbalance, and increased oxidative stress, all of which are known to elevate cardiovascular risk [11]. Furthermore, irregular meal patterns, reduced leisure-time physical activity, and high job strain exacerbate the likelihood of developing metabolic disorders and other chronic conditions [10,11].

Several studies have reported that PO exhibit higher prevalence rates of obesity, hypertension, dyslipidaemia, and MetS compared with the general population [10,12]. For example, Hartley et al. [8] reported a higher prevalence of MetS among American PO, compared to other employed adults. According to Yates et al. [13], similar findings have been reported across Europe, with prevalence rates ranging from 10% to 33%, depending on the occupational context and lifestyle patterns. These data reinforce the notion that the law enforcement profession should be considered a high-risk occupational category for cardiovascular and metabolic diseases.

Beyond biological and behavioural determinants, the psychosocial environment of policing also plays a critical role in shaping health outcomes [9,10]. Chronic exposure to stress has been linked not only to increased blood pressure and impaired glucose regulation but also to reduced mental health and lower perceived quality of life [9,14]. Health-Related Quality of Life (HRQoL) is a multidimensional construct encompassing physical, psychological, and social domains of well-being [15,16]. It is considered an essential indicator of overall health status. It has gained importance in occupational health research, particularly in professions characterised by chronic stress and high physical or emotional demands [15,16]. Among POs, lower HRQoL scores have been associated with greater stress, burnout, sleep problems, and metabolic abnormalities, emphasising the bidirectional relationship between physical and mental health [17,18].

In this context, sex and occupational function are important determinants of both metabolic health and perceived quality of life. Several studies have shown that male PO tend to present higher cardiometabolic risk profiles, mainly due to greater tobacco use, higher body mass index (BMI), and elevated blood pressure. In contrast, female PO frequently report poorer quality of life, particularly in physical and emotional health domains [11,14]. Additionally, job function within the police force (operational/outdoor *versus* administrative/indoor) has been identified as a differentiating factor in health outcomes, i.e., POs working primarily indoors in administrative or support roles are often more sedentary, which can exacerbate metabolic dysfunction [13,19]. In contrast, those in operational functions tend to maintain higher physical activity levels, potentially mitigating some cardiovascular risks [13,19].

Despite increasing international evidence regarding cardiometabolic risk among POs, research in the Portuguese context remains limited [14], and, to the best of our knowledge, no studies have specifically examined this issue in the Autonomous Region of Madeira. In fact, little is known about the cardiometabolic profile of POs in this Portuguese Region.

Furthermore, this region represents a distinctive context for occupational health research due to its insular geography, relatively small and geographically concentrated population, and the organisational characteristics of its police force. Policing in island territories may involve operational routines, potentially influencing work-related stress, physical activity patterns, and cardiometabolic health. Therefore, examining MetS, cardiovascular risk, and HRQoL among POs in Madeira may provide valuable evidence to inform targeted occupational health strategies within this regional policing context.

Therefore, this study aimed to determine the prevalence of MetS, estimate 10-year cardiovascular risk using the Framingham Risk Score, evaluate HRQoL, and explore potential differences according to sex and duty (indoor *versus* outdoor) among POs in the Autonomous Region of Madeira.

By combining objective clinical and biochemical measures with subjective health perceptions, this study provides an integrative perspective on the physical and psychological well-being of POs. The findings are expected to contribute to the growing body of evidence on occupational health in law enforcement and to support the implementation of preventive programs, such as regular screening, smoking cessation initiatives, and workplace physical activity interventions, within the PSP framework in Madeira.

## 2. Materials and Methods

### 2.1. Study Design

A cross-sectional observational study was conducted to evaluate the prevalence of MetS, cardiovascular risk, and HRQoL among POs in the Autonomous Region of Madeira, Portugal.

### 2.2. Ethical Considerations

The study was conducted in accordance with the Declaration of Helsinki and followed the ethical and methodological standards for human research. Ethical approval was obtained from the Instituto Superior de Ciências Policiais e Segurança Interna (ISCPSI) ethics committee (Protocol code: VICCDP/PROJETOS/TB/1-2025; Date: 25 July 2025).

The study was conducted in collaboration with the Occupational Health Department of PSP-Madeira, and participants were recruited during routine occupational health evaluations. Inclusion criteria were: (i) age ≥ 30 years; (ii) active duty at the time of data collection; and (iii) absence of acute illness or injury that could interfere with participation. POs on medical leave or with incomplete data were excluded from the analysis.

Participation was voluntary, confidentiality and anonymity were fully guaranteed, and all participants provided written informed consent before participation.

### 2.3. Participants

Of the 742 eligible POs from the Madeira Regional Command of the PSP, 109 agreed to participate in the study (response rate: 14.7%). The sample included 88 male POs (80.7%) and 21 female POs (19.3%), aged 30–59 years. POs were classified by job function: (i) 48.6% with indoor/support roles (n = 53; male, n = 35; female, n = 18), and (ii) 51.4% with outdoor/operational roles (n = 56; male, n = 53; female, n = 3).

The minimum sample size for this cross-sectional study was estimated assuming a 95% confidence level and an 8% margin of error. Based on an expected prevalence of MetS of 28.2% and a finite population of 742 eligible POs, the minimum required sample size after applying the finite population correction was 105 participants.

### 2.4. Procedures

Data collection was carried out in collaboration with the Occupational Health Services of PSP-Madeira. All assessments were performed in the morning, under standardised conditions, after a 12 h fasting period and abstinence from caffeine, alcohol, and vigorous exercise in the previous 24 h.

The evaluation protocol included six main components: (i) anthropometric assessment; (ii) blood pressure measurement; (iii) biochemical analysis; (iv) diagnosis of MetS; (v) Framingham cardiovascular risk assessment; and (vi) HRQoL.

Anthropometric assessment included: (i) body height, measured using a portable stadiometer (SECA 213, SECA GmbH & Co. KG, Hamburg, Germany) with PO standing barefoot, with their heels together, and their head aligned in the Frankfurt plane; (ii) body weight, obtained using a calibrated mechanical scale (SECA 761, Hamburg, Germany), and BMI was calculated as weight divided by height squared (kg/m^2^); and (iii) waist circumference, measured with a non-elastic flexible tape (SECA 201, Hamburg, Germany) at the midpoint between the lower margin of the last rib and the iliac crest, after normal expiration. All anthropometric procedures followed international standards [20].

To assess blood pressure, systolic blood pressure (treated/untreated) was measured using a validated automatic monitor (Omron M2, Omron Healthcare, Kyoto, Japan) in a seated position, with the arm supported at heart level, after a minimum of 10 minutes’ rest in a quiet environment. Appropriate cuff sizes were selected based on arm circumference.

To evaluate biochemical parameters, venous blood samples (~10 mL) were collected by trained clinical technicians after a 12 h overnight fast. Analyses were performed at a certified clinical laboratory following internal and external quality control protocols in accordance with international standards [21,22]. Serum glucose, total cholesterol (TC), high-density lipoprotein cholesterol (HDL-C), and triglycerides (TG) were determined using colorimetric enzymatic methods [23]. Low-density lipoprotein cholesterol (LDL-C) was calculated using the Friedewald formula: LDL-C = TC − (HDL-C + TG/5) [24].

MetS was defined according to the National Cholesterol Education Program—Adult Treatment Panel III (NCEP ATP III) criteria [25]. The diagnosis was confirmed when three or more of the following five criteria were present:

- Abdominal obesity: waist circumference >102 cm in men or >88 cm in women;
- Hypertriglyceridemia: triglycerides ≥ 150 mg/dL;
- Low HDL-C: <40 mg/dL in men or <50 mg/dL in women;
- Elevated blood pressure: ≥130/85 mmHg or use of antihypertensive medication;
- Elevated fasting glucose: ≥100 mg/dL.

Framingham cardiovascular risk was estimated using the Framingham Risk Score [6,26], which predicts the 10-year probability of developing a major cardiovascular event. The score incorporates: (i) age; (ii) sex; (iii) total cholesterol; (iv) HDL-C; (v) systolic and diastolic blood pressure; (vi) diabetes status; and (vii) smoking status. POs were classified as: (i) low risk (<10%); (ii) moderate risk (10–20%); or (iii) high risk (>20%).

HRQoL was assessed using the Short Form Health Survey (SF-36v2) [16,27]. The validated Portuguese version [28,29] was used. The SF-36v2 comprises 36 items grouped into eight dimensions: (i) physical function; (ii) physical performance; (iii) body pain; (iv) general health; (v) vitality; (vi) social function; (vii) emotional performance; and (viii) mental health. Scores for each dimension range from 0 to 100, with higher scores indicating better perceived health. Two summary measures were calculated: (i) the Physical Component Summary; and (ii) the Mental Component Summary.

### 2.5. Statistical Analysis

All statistical analyses were performed using JASP software (version 0.18.3, University of Amsterdam, The Netherlands). Descriptive statistics were calculated for demographic, clinical, and job variables. Continuous variables are presented as mean (M) ± standard deviation (SD) and median with interquartile range (IQR), while categorical variables are expressed as absolute (n) and relative (%) frequencies. Normality of the distributions was assessed using the Shapiro–Wilk test before selecting the statistical procedures.

Because several variables did not meet normality assumptions and group sizes were unbalanced, non-parametric tests were applied. Therefore, (i) the Mann–Whitney U test was used to compare continuous variables between groups, and the effect size is given by the rank biserial correlation (*r*) interpreted as small, medium and large; (ii) the Chi-square test was used for categorical variables, and effect sizes for categorical comparisons were estimated using Cramér’s V, interpreted as small (0.10), medium (0.30), and large (0.50) associations.

To evaluate independent associations between professional duties and cardiometabolic outcomes among male POs, multivariable regression models were conducted: (i) Logistic regression analysis was used to examine factors associated with MetS, and the dependent variable was the presence of MetS (yes/no), and independent variables included duty type (indoor *versus* outdoor), age, BMI, and smoking status; and (ii) Multiple linear regression analysis was performed to assess predictors of the Framingham 10-year cardiovascular risk score (the Framingham risk score was treated as a continuous dependent variable), and the same set of independent variables (professional duty type, age, BMI, and smoking status) were included in the model to control for potential confounding factors. Odds ratios (OR) with 95% confidence intervals (CI) were reported for logistic regression analyses, while beta coefficients (β), standard errors (SE), and 95% confidence intervals were reported for linear regression models. Model goodness-of-fit was assessed using pseudo-*R*^2^ for logistic regression and *R*^2^ for linear regression models. Statistical significance was set at *p* < 0.05.

## 3. Results

**Figure 1:**
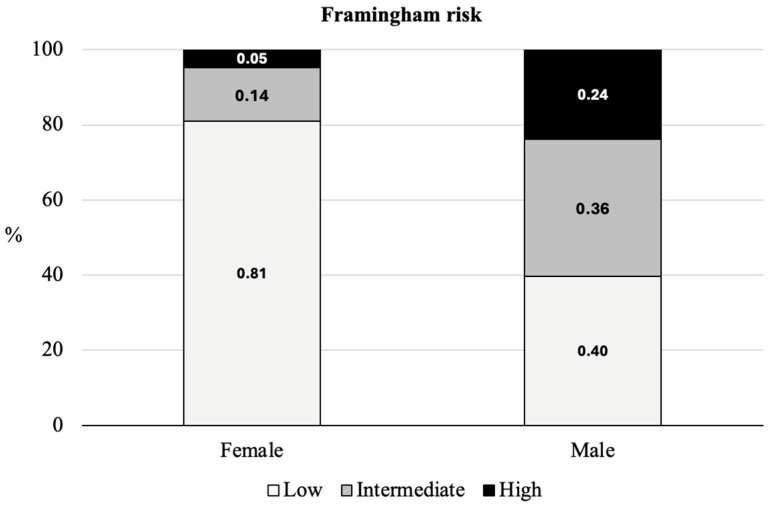
Risk of cardiovascular events in 10 years according to sex.

**Figure 2:**
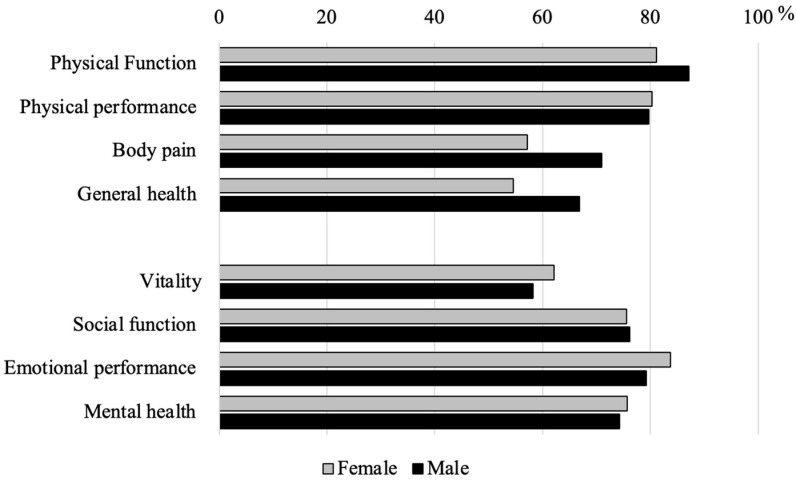
Assessment of Quality-of-Life dimensions according to sex.

Male POs were significantly taller and heavier than women (*p* < 0.01). They showed higher 10-year Framingham Risk Scores and higher scores in the bodily pain and general health dimensions than female POs. Smoking status and Framingham risk category differed significantly according to sex. The results are presented in Table 1 and illustrated in Figure 1 and Figure 2.

Due to the very small number of female POs performing outdoor duties (n = 3), subgroup analyses stratified by both sex and job function were not performed. Therefore, comparisons by job function were conducted among male POs, who comprised the majority of the sample, and it was observed that male POs performing indoor duties had significantly higher 10-year Framingham risk scores than those performing outdoor duties (*p* < 0.05). The results are presented in Table 2.

Multivariable regression analyses were conducted among male POs to explore the independent association between occupational duties and cardiometabolic outcomes.

In the logistic regression model (Table 3): (i) indoor duties were not independently associated with MetS after adjustment for age, BMI, and smoking status (*OR* = 0.78, 95% CI: 0.26–2.34, *p* = 0.65); (ii) increasing age was significantly associated with higher odds of MetS (*OR* = 1.13 per year, 95% CI: 1.03–1.24, *p* = 0.01); (iii) higher BMI was independently associated with MetS (*OR* = 1.20 per kg/m^2^, 95% CI: 1.01–1.42, *p* = 0.04); and (iv) smoking status also showed a significant association, with smokers presenting markedly higher odds of MetS (*OR* = 4.46, 95% CI: 1.29–15.38, *p* = 0.02).

In the multiple linear regression model assessing determinants of the Framingham 10-year cardiovascular risk score (Table 4): (i) indoor duties were not significantly associated with cardiovascular risk (*β* = 0.84, 95% CI: −1.29 to 2.97, *p* = 0.44); (ii) age was strongly associated with higher cardiovascular risk (*β* = 0.56, 95% CI: 0.45–0.74, *p* < 0.01); (iii) smoking was also a significant predictor, increasing the Framingham risk score by approximately 8.55 percentage points (95% CI: 6.07–11.02, *p* < 0.01); and (iv) BMI showed a positive but non-significant association with cardiovascular risk (*β* = 0.23, *p* = 0.12). The model explained a substantial proportion of the variance in Framingham risk (*R*^2^ = 0.62).

## 4. Discussion

The present study identified a 28.4% prevalence of MetS among POs from the Autonomous Region of Madeira. This value aligns with international studies of police populations, although prevalence estimates vary by methodological approach and regional context. For example, Tharkar et al. [12] reported that the prevalence of metabolic syndrome was significantly higher among Indian POs compared to GP (57.3% *vs.* 28.2%), while Yates et al. [13] observed MetS in 20% of British POs performing operational duties (outdoor) and in 13.6% of those in non-operational roles (indoor). Studies conducted in North American police cohorts frequently report higher prevalence rates, such as 26.7% reported by Hartley et al. (2011), compared with 18.7% in the general working population [30]. Overall, previous research suggests that the prevalence of MetS in police populations generally ranges between 26% to 33% [10], placing the prevalence observed in the present study within the expected range for this group of POs.

Contrary to much of the existing literature, no statistically significant differences in MetS prevalence were observed between male and female POs. This contrasts with studies indicating higher rates among men [9]. Two factors may explain this discrepancy: (i) the relatively small number of female POs, which limits statistical power; and/or (ii) the potential homogeneity in work conditions within PSP-Madeira, which may attenuate gender disparities typically observed in larger or more heterogeneous police forces. Therefore, these findings should be interpreted cautiously.

Regarding cardiovascular risk, approximately one in five POs (20.2%) presented a high 10-year risk of cardiovascular events according to the Framingham Risk Score. This proportion exceeds the 9.6% reported in German POs [19] and exceeds the estimated prevalence of elevated cardiovascular risk in the general Portuguese population [31]. However, comparisons with national estimates should be interpreted cautiously, as different risk prediction models are used. While Portuguese population estimates are often based on the SCORE system, which predicts fatal cardiovascular events, the Framingham model incorporates both fatal and non-fatal outcomes [6,7].

Nevertheless, these results reinforce the evidence that policing represents an occupational group exposed to increased cardiovascular risk, largely due to chronic occupational stress, irregular working schedules, and sleep disruption [9]. The higher proportion of high-risk profiles among male POs is consistent with previous findings [32] and appears to be partly explained by the presence of smoking exclusively among men in the present sample and by the weighting of male sex in the Framingham algorithm.

A particularly relevant finding concerns occupational function. POs performing indoor administrative or support duties presented significantly higher Framingham risk scores than those engaged in outdoor operational roles. This difference may reflect variations in occupational physical demands, as administrative functions are generally associated with more sedentary work patterns.

It is important to note that female analyses combining job functions (indoor *versus* outdoor) were not conducted due to the extremely small number of female POs assigned to outdoor operational duties (n = 3). Statistical comparisons across small subgroups may yield unreliable estimates and were therefore avoided.

In the present study, duty type was not independently associated with MetS or estimated cardiovascular risk after adjustment for major cardiometabolic risk factors among male POs. Instead, traditional cardiovascular risk factors, particularly age, BMI, and smoking, were the main determinants of MetS. In contrast, age and smoking were the strongest predictors of the Framingham cardiovascular risk score. However, the lack of an independent association between indoor duties and cardiometabolic outcomes observed in the present study suggests that occupational assignment alone may not fully explain the increased cardiovascular risk reported in police populations. Instead, individual-level risk factors such as age, BMI, and smoking appear to play a more prominent role.

Overall, the findings suggested that POs in Madeira represent a population with relevant cardiometabolic and psychosocial health vulnerabilities. These risks appear to result from a combination of individual behaviours, such as smoking and elevated BMI, and job characteristics, including sedentary work patterns and exposure to chronic job stress.

In fact, previous studies have similarly reported that POs in administrative roles tend to exhibit higher levels of abdominal obesity and dyslipidaemia compared with their operational personnel [13,19]. However, because physical activity levels were not directly measured in this study, the mechanisms underlying these differences remain speculative.

Regarding HRQoL, male POs reported significantly lower levels of bodily pain and better general health than female POs. This pattern is consistent with previous literature indicating that female POs often report lower HRQoL scores in domains related to physical health and pain [11]. In the Portuguese policing context, similar findings were reported by Faria et al. (2024), who observed lower HRQoL scores among female POs in several physical and psychosocial domains [14]. Nevertheless, potential gender-related reporting differences should be considered, as women may report health symptoms more consistently than men [33].

An additional factor that may contribute to the cardiometabolic profile observed in this group of POs is occupational factors commonly present in policing, particularly shift work and variations in physical activity levels. PO frequently perform rotating or irregular shifts, which are associated with circadian rhythm disruption, sleep deprivation, and hormonal changes that may adversely affect metabolic regulation and cardiovascular health [10,11]. Previous research has shown that shift work is associated with higher rates of obesity, hypertension, and MetS in law enforcement personnel [10]. In addition, levels of physical activity may vary considerably by job function, with POs assigned to administrative or indoor roles often exhibiting lower daily energy expenditure than those engaged in operational duties [13]. Recent European studies have highlighted the protective role of higher physical activity and cardiorespiratory fitness in reducing cardiovascular risk among POs and other occupational groups with demanding work conditions [13,19]. In other words, research conducted in European police cohorts has demonstrated that higher cardiorespiratory fitness is associated with more favourable cardiovascular risk profiles and lower prevalence of metabolic abnormalities [19]. Because physical activity levels were not measured, the mechanisms underlying these differences remain speculative.

The practical implications of these results are clear and directly applicable to occupational health management within the PSP, including the implementation of (i) smoking cessation programs, particularly targeting male POs; (ii) structured physical activity within the workplace, especially for administrative/support staff; (iii) regular and systematic screening of metabolic risk factors (blood pressure, lipid profile, glucose, waist circumference); and (iv) gender-specific interventions addressing musculoskeletal health and psychological well-being among female POs.

Several limitations should be acknowledged: (i) the cross-sectional design prevents causal inferences regarding the relationship observed; (ii) the study was conducted in a regional police population, which may limit the generalizability of the findings to other policing contexts; (iii) some variables were based on self-reported information, which may introduce reporting bias; (iv) the relatively small number of female POs limited the possibility of conducting detailed subgroup analyses; (v) although the response rate was relatively low, the final sample size met the minimum estimated requirement for the specified confidence level and margin of error (nevertheless, the findings should be interpreted with caution due to the potential for selection bias inherent to voluntary participation); and (vi) the absence of several relevant occupational and behavioural variables known to influence cardiometabolic risk in police populations (physical activity, shift work patterns, years of service, dietary habits, alcohol consumption, and perceived occupational stress). In accordance, future studies should incorporate these dimensions, preferably using objective or validated measures, and adopt longitudinal designs to better understand the progression of cardiometabolic risk factors in police populations.

## 5. Conclusions

This study presents the first comprehensive characterisation of metabolic health and cardiovascular risk among POs in the Autonomous Region of Madeira.

The findings indicate that the prevalence of MetS falls within international ranges, reinforcing that POs constitute a professional group with elevated cardiometabolic vulnerability. The proportion of POs classified as high cardiovascular risk according to the Framingham model was notably higher than that reported for the general Portuguese population, highlighting a specific susceptibility within this professional group.

Although no sex differences in MetS prevalence were identified, contrary to most international evidence, this pattern may reflect the relatively homogeneous working conditions shared by male and female POs in PSP-Madeira.

Importantly, male POs assigned to indoor administrative duties demonstrated higher cardiovascular risk than those performing outdoor operational functions, suggesting that sedentarism plays a meaningful role in shaping cardiometabolic health. Although duty type was not independently associated with cardiometabolic outcomes after adjustment for traditional risk factors, age, body mass index, and smoking emerged as key determinants of MetS. In contrast, age and smoking were the strongest predictors of estimated cardiovascular risk.

Regarding HRQoL, male POs reported less bodily pain and better general health than female POs. At the same time, female POs expressed more negative perceptions, possibly reflecting gender-specific patterns in symptom perception and reporting.

In sum, beyond confirming trends described in the literature, the study highlights the unexpected absence of sex disparities in metabolic risk and the differentiated impact of job function. These results underscore that police work in Madeira presents a relevant burden of cardiovascular risk factors that require targeted occupational health interventions within police organisations, including regular cardiometabolic screening, promoting physical activity, and implementing workplace health programs.

## Figures and Tables

**Table 1:** Anthropometric attributes, blood pressure, blood biochemical parameters, metabolic syndrome, Framingham risk, and quality of life of female and male POs.

		Female		Male		Total	Statistics	*p*-Value	ES
		M ± SD	IQR	M ± SD	IQR	M ± SD			
Age (years)		50.24 ± 6.11	10.00	47.13 ± 7.57	9.25	47.73 ± 7.39	1117.50	0.14	0.21
Anthropometric Assessment ^a^									
Height (m)		1.64 ± 0.04	7.00	1.75 ± 0.12	9.25	1.73 ± 0.12	117.50	<0.01	−0.87
Weight (kg)		74.57 ± 10.47	15.00	86.94 ± 11.73	15.25	84.56 ± 12.46	392.50	<0.01	−0.58
Waist circumference (cm)		90.19 ± 10.89	17.00	95.78 ± 9.30	13.25	94.71 ± 9.83	713.50	0.11	−0.23
Body mass index (kg/m^2^)		27.67 ± 4.01	5.24	27.93 ± 3.78	3.47	27.88 ± 3.81	925.00	1.00	<0.01
Blood Pressure Assessment ^a^									
Systolic BP (mmHg)		124.86 ± 16.45	14.00	130.24 ± 16.70	17.25	129.20 ± 16.71	782.50	0.28	−0.15
Diastolic BP (mmHg)		84.48 ± 12.36	15.00	83.17 ± 8.66	9.25	83.42 ± 9.43	915.00	0.95	−0.01
Blood Biochemical Parameters ^a^									
Glucose (mg/dL)		91.52 ± 13.66	11.00	95.49 ± 17.98	15.00	94.73 ± 17.25	746.50	0.17	−0.19
HDL cholesterol (mg/dL)		59.19 ± 20.76	32.00	50.72 ± 14.60	14.25	52.35 ± 16.21	1125.00	0.12	0.22
LDL cholesterol (mg/dL)		106.67 ± 35.63	46.00	109.30 ± 35.88	51.25	108.79 ± 35.68	859.00	0.62	−0.07
Total cholesterol (mg/dL)		189.10 ± 37.53	56.00	183.08 ± 38.73	47.00	184.24 ± 38.40	1016.50	0.48	0.10
Triglycerides (mg/dL)		120.67 ± 43.82	35.00	133.74 ± 80.93	96.00	131.22 ± 75.22	915.00	0.95	−0.01
Risk Assessment (Metabolic Syndrome and Framingham)									
Smoking ^b^	Yes	0 (0.0)		19 (21.6)		19 (17.4)	5.49	0.02	0.22
	No	21 (100.0)		69 (78.4)		90 (82.6)			
Diabetes ^b^	Yes	2 (9.5)		3 (3.4)		5 (4.6)	1.45	0.23	0.12
	No	19 (90.5)		85 (96.6)		104 (95.4)			
Hypertension ^b^	Yes	2 (9.5)		15 (17.0)		17 (15.6)	0.73	0.39	0.08
	No	19 (90.5)		73 (83.0)		92 (84.4)			
Metabolic syndrome ^b^	Present	6 (28.6)		25 (28.4)		31 (28.4)	2.195 × 10^−4^	0.99	<0.01
	Absent	15 (71.4)		63 (71.6)		78 (71.6)			
Framingham risk ^b^	High	1 (4.8)		21 (23.8)		22 (20.2)	11.67	<0.01	0.33
	Moderate	3 (14.3)		32 (36.4)		35 (32.1)			
	Low	17 (80.9)		35 (39.8)		52 (47.7)			
10-yr Framingham risk (%) ^a^		5.24 ± 4.61	3.00	12.35 ± 7.55	10.00	10.98 ± 7.60	348.00	<0.01	−0.62
Quality of Life Assessment ^a^									
Physical function (%)		81.19 ± 20.49	20.00	87.16 ± 20.10	20.00	86.01 ± 20.22	682.50	0.05	−0.261
Physical performance (%)		80.36 ± 17.49	25.00	79.76 ± 22.86	31.25	79.87 ± 21.86	868.50	0.67	−0.060
Body pain (%)		57.14 ± 23.28	33.00	71.05 ± 24.75	39.00	68.37 ± 24.98	606.00	0.01	−0.344
General health (%)		54.62 ± 15.23	25.00	66.92 ± 20.08	30.00	64.55 ± 19.79	590.50	0.01	−0.361
Vitality (%)		62.20 ± 15.37	18.75	58.24 ± 20.40	31.25	59.00 ± 19.53	1052.50	0.32	0.139
Social function (%)		75.60 ± 19.15	25.00	76.14 ± 22.56	37.50	76.03 ± 21.86	867.50	0.66	−0.061
Emotional performance (%)		83.73 ± 14.55	25.00	79.26 ± 23.67	33.33	80.12 ± 22.22	952.00	0.83	0.030
Mental health (%)		75.71 ± 13.81	15.00	74.26 ± 19.05	26.25	74.54 ± 18.11	904.50	0.88	−0.021

Legend: ^a^, mean ± standard deviation and Mann–Whitney test results (test statistic and *p*-value); ^b^, n (%) and Chi-Square test results (test statistic, degrees of freedom and *p*-value); ES, Effect Size (rank biserial correlation (*r*) for continuous variables; Cramér’s V for categorical comparisons were estimated using).

**Table 2:** Anthropometric attributes, blood pressure, blood biochemical parameters, risk of metabolic syndrome, Framingham risk, and quality of life of male POs performing duties inside and outside facilities.

		Indoor		Outdoor		Statistics	*p*-Value	ES
		M ± SD	IQR	M ± SD	IQR			
Age (years) ^a^		49.09 ± 7.16	10.50	45.83 ± 7.62	8.00	697.50	0.05	−0.25
Anthropometric Assessment ^a^								
Height (m)		177.17 ± 6.41	10.00	173.25 ± 14.19	8.00	732.50	0.10	−0.21
Weight (kg)		88.42 ± 14.39	18.00	85.96 ± 9.61	10.00	801.00	0.28	−0.14
Waist circumference (cm)		97.31 ± 10.52	11.50	94.77 ± 8.35	12.00	794.00	0.26	−0.14
Body mass index (kg/m^2^)		28.17 ± 4.18	5.20	27.78 ± 3.52	3.42	885.50	0.72	−0.05
Blood Pressure Assessment ^a^								
Systolic BP (mmHg)		132.91 ± 18.40	23.50	128.47 ± 15.40	13.00	802.00	0.29	−0.14
Diastolic BP (mmHg)		83.49 ± 10.70	14.50	82.96 ± 7.10	7.00	877.00	0.67	−0.05
Blood Biochemical Parameters ^a^								
Glucose (mg/dL)		93.54 ± 10.81	14.00	96.77 ± 21.45	15.00	909.00	0.88	−0.02
HDL cholesterol (mg/dL)		52.54 ± 19.19	18.00	49.51 ± 10.60	11.00	880.00	0.69	−0.05
LDL cholesterol (mg/dL)		113.34 ± 34.92	54.50	106.62 ± 36.58	49.00	803.00	0.29	−0.13
Total cholesterol (mg/dL)		190.51 ± 35.10	44.00	178.17 ± 40.53	39.00	713.50	0.07	−0.23
Triglycerides (mg/dL)		136.63 ± 96.11	108.50	131.83 ± 70.05	90.00	945.00	0.89	0.02
Risk Assessment (Metabolic Syndrome and Framingham)								
Smoking ^b^	Yes	9 (25.7)		10 (18.9)		0.58	0.45	0.08
	No	26 (74.3)		43 (81.1)				
Diabetes ^b^	Yes	2 (5.7)		1 (1.9)		0.94	0.33	0.10
	No	33 (94.3)		52 (98.1)				
Hypertension ^b^	Yes	7 (20.0)		8 (15.1)		0.36	0.55	0.06
	No	28 (80.0)		45 (84.9)				
Metabolic syndrome ^b^	Present	11 (31.4)		14 (26.4)		0.26	0.61	0.05
	Absent	24 (68.6)		39 (73.6)				
Framingham risk ^b^	High	12 (34.3)		9 (17.0)		3.73	0.16	
	Moderate	11 (31.4)		20 (37.7)				0.21
	Low	12 (34.3)		24 (45.3)				
10-year Framingham risk (%) ^a^		14.43 ± 8.15		10.98 ± 6.86		693.50	0.05	−0.25
Quality of Life Assessment ^a^								
Physical function (%)		86.86 ± 18.83	20.00	87.36 ± 21.07	15.00	969.00	0.71	0.05
Physical performance (%)		83.39 ± 23.38	25.00	77.36 ± 22.41	37.50	748.50	0.12	−0.19
Body pain (%)		72.49 ± 23.63	33.00	70.09 ± 25.64	49.00	895.50	0.78	−0.04
General health (%)		69.60 ± 19.22	26.50	65.14 ± 20.62	32.00	804.00	0.29	−0.03
Vitality (%)		57.68 ± 20.90	31.25	58.61 ± 20.26	31.25	937.00	0.94	0.01
Social function (%)		78.57 ± 23.60	25.00	74.53 ± 21.92	25.00	805.50	0.29	−0.13
Emotional performance (%)		80.24 ± 22.96	33.33	78.62 ± 24.32	41.67	894.50	0.77	−0.04
Mental health (%)		74.57 ± 19.57	22.50	74.06 ± 18.89	25.00	896.50	0.79	−0.03

Legend: ^a^, mean ± standard deviation and Mann–Whitney test results (test statistic and *p*-value); ^b^, n (%) and Chi-Square test results (test statistic, degrees of freedom and *p*-value); ES, Effect Size (rank biserial correlation (*r*) for continuous variables; Cramér’s V for categorical comparisons were estimated using).

**Table 3:** Multivariable logistic regression for metabolic syndrome in male POs.

Variables	*OR*	95% CI	*p*-Value	Model Statistics
Indoor duties (*vs.* outdoor)	0.78	0.26–2.34	0.65	Pseudo *R*^2^ = 0.21 Likelihood ratio test *p* < 0.01
Age (per year)	1.13	1.03–1.24	0.01	
Body mass index (kg/m^2^)	1.20	1.01–1.42	0.04	
Smoking (yes *vs.* no)	4.46	1.29–15.38	0.02	

**Table 4:** Multiple linear regression for Framingham 10-year cardiovascular risk score in male POs.

Variables	*β*	SE	95% CI	*p*-Value	Model Statistics
Indoor duties (*vs.* outdoor)	0.84	1.07	−1.29–3.00	0.44	*R*^2^ = 0.62 Adjusted *R*^2^ = 0.60 *F*(4, 83) = 33.51, *p* < 0.01
Age (per year)	0.60	0.07	0.45–0.74	<0.01	
Body mass index (kg/m^2^)	0.23	0.14	−0.06–0.51	0.12	
Smoking (yes *vs.* no)	8.55	1.25	6.07–11.02	<0.01	

## Data Availability

The data presented in this study are available on reasonable request from the corresponding author. The data are not publicly available due to ethical and privacy restrictions related to occupational health data.

## Abbreviations

The following abbreviations are used in this manuscript:

BMI	Body mass index
BP	Blood Pressure
CI	Confidence intervals
CVD	Cardiovascular Disease
ES	Effect Size
HDL-C	High-Density Lipoprotein Cholesterol
HRQoL	Health-Related Quality of Life
IQR	Interquartile range
ISCPSI	Instituto Superior de Ciências Policiais e Segurança Interna
LDL-C	Low-Density Lipoprotein Cholesterol
M	Mean
MetS	Metabolic Syndrome
NCEP-ATP III	National Cholesterol Education Program Adult Treatment Panel III
OR	Odds ratios
PO	Police Officer
Pos	Police Officers
PSP	Polícia de Segurança Pública
R	rank biserial correlation
SD	Standard deviation
SE	Standard errors
SF-36v2	Short Form Health Survey
SF-36v2	Short Form Health Survey, Version 2
TC	Total cholesterol
TG	Triglycerides
Β	Beta coefficients
